# Meetings of Societies

**Published:** 1921-06

**Authors:** 


### MEETINGS OF SOCIETIES.

On Wednesday the 12th of January, 1921, the Bristol Medico-Chirurgical Society held a meeting which was devoted to a discussion on **Psychotherapy**. Dr. L. E. V. Every-Clayton was in the Chair.

Dr. R. G. Gordon opened the discussion. (Paper published in full on p. 11).

Professor Lloyd Morgan pleaded for closer union between academic and clinical psychology, referring to an article recently published by him in favour of the teaching of psychology to medical students. He asked the clinical psychologists to " nail their colours to the mast," and to state to which school of psychology they adhered.

Dr. J. R. R. Trist spoke of the work of a neurological clinic of the Ministry of Pensions, and illustrated the impossibility of separating psychotherapy from other forms of treatment by data regarding the incidence of tuberculosis among neurasthenic ex-service men.

Dr. H. H. Carleton referred all conflict to a struggle between the egotistical instincts on the one hand and the higher, more recently acquired altruistic motives on the other, and alluded to the " herd instinct."

Dr. O. H. Woodcock confessed himself an adherent of the animistic school of psychology. As a means of preventing psychical disorders he declared his belief in educating the individual to a better understanding of himself and his relations to the cosmic order.

Dr. J. R. Charles criticised the philosophy on which modern psychotherapy is founded, pointing out that " conflict " is not merely inevitable but actually necessary as a means of education for the individual and the race. He deprecated the use of hypnosis as a therapeutic measure.

Dr. C. F. Coombs alluded to the disturbances of visceral function that arise from the psycho-neuroses, and also to the part that similar disturbances often play in aggravating the symptoms of organic disease.

Dr. E. T. Glenny attacked the work of the psycho-analysts, particularly on the grounds that it was falling into the hands of practitioners with inadequate experience of organic disease, and that undue insistence on sexual motives was discrediting the profession in the eyes of the public.

Major A. E. Lister, I.M.S., described the case of an Indian soldier and his recovery from a psycho-neurosis by means of auto-suggestion.

To these comments and criticisms Dr. R. G. Gordon replied.

---

A meeting of the Bristol Medico-Chirurgical Society held on February 9th was devoted to discussion of the subject of **Shortness of Breath,** opened by the President, Dr. L. E. V. Every-Clayton, who gave a brief review of the physiology of respiration, and divided the causes of shortness of breath into inhibitions of respiratory movement, occlusion of air passages, and changes in the composition and circulation of the blood in the lungs. Examples of each group were given, and a number of propositions made by way of furnishing a basis for discussion.

Dr. J. R. Charles demonstrated and explained Fridericia's apparatus for measuring the carbon dioxide content of the alveolar air. He showed that dyspnœa was caused whenever an increase of the circulating acids, volatile or otherwise, stimulated the respiratory centre to increased effort, and instanced the air-hunger of diabetic coma and of salicylate poisoning, as well as the dyspnœa accompanying muscular effort and provoked by excess of lactic acid. He drew attention to some of the discrepancies to be observed in pulmonary disease, where a small lesion such as an infarct might cause more dyspnœa than was often encountered in much grosser lesions of the lung.

Professor F. H. Edgeworth discriminated between subjective breathlessness and quickened respiration, and instanced "Cheyne-Stokes" breathing as a condition in which the patient had little or no discomfort. In pneumonia, with a respiration rate of 40 per minute, there might be no subjective dyspnœa, but a patient with bronchiolitis breathing at the same speed would feel very short of breath. He also alluded to hysterical quickness of breathing.

DR. J. O. SYMES mentioned some of the conditions in which it was difficult to explain the dyspnœa present, remarking that in cases of cardiac disease without murmurs it was apt to be a prominent symptom, and that in emphysema of the lungs it was often difficult to determine whether the shortness of breath was due to the emphysema itself or to the myocardial degeneration and the obesity which so often accompanied it. He also spoke of the dyspnœa of hyperthyroidism and of mediastinal tumour.

DR. C. F. COOMBS gave a brief account of the origin of dyspnœa in cardiac disease, speaking particularly of conditions of deficient aëration due to right heart failure, and also summarising recent work on the part played by acidosis in the causation of " cardiac asthma " and allied disorders of respiration. He pointed out that even in the presence of organic disease of the heart the nervous element had to be considered.

DR. R. G. GORDON said that he was impressed by the part played by the endocrine glands and the autonomic nervous system in the production of dyspnœa, and instanced the effect of emotion and of adrenalin in relieving asthma.

DR. E. H. E. STACK asked how it was that children with evidence of acidosis might nevertheless be free from dyspnœa.

DR. CECIL CLARKE spoke of the disproportionate dyspnœa of early tuberculosis of the lung, which he thought might be due to the action of bacterial toxins, a proposition which he supported by quoting the fact that pneumococcal infection of tissues other than the lung can quicken the respiration.

DR. J. A. NIXON described his war experience of gunshot wounds of the chest. He stated that in these the dyspnœa was not solely determined by the extent of lung injured or to the bulk of intrapleural extravasation. Infection of the blood within the pleural cavity quickened respiration. He also quoted examples to show that displacement of the mediastinum directly, and irritation of the pleura reflexly, had great influence on the degree of dyspnœa.

DR. H. H. CARLETON confirmed what was said as to the importance of the pleural reflex from his experiences in the artificial induction of pneumothorax.

DR. O. H. WOODCOCK gave a brief account of a case of hysterical dyspnœa due to a psycho-neurosis.

DR. R. C. CLARKE had also noted that infection of the contents of a hæmothorax quickened the respiration.

The President briefly summed up, congratulating the Society on having brought forward so much that was interesting and instructive.

